# Post-processing T-patterns Using External Tools From a Mixed Method Perspective

**DOI:** 10.3389/fpsyg.2019.01680

**Published:** 2019-07-24

**Authors:** István Szekrényes

**Affiliations:** Institute of Philosophy, University of Debrecen, Debrecen, Hungary

**Keywords:** theme, mixed methods, observation, annotation, post-analysis

## Abstract

Researchers of behavioral science often work with time-aligned annotation data based on video and/or audio recordings. Various platforms are available to process these data, offering various kinds and ways of data analysis. It often happens that one would wish to use one platform for a certain kind of analysis, and another platform for another kind. It may also happen that one would keep the results of the first analysis and continue processing the data using another platform—all as a chain of analyses on the way to discovery. When it comes to T-pattern analysis, the task of further analyzing already identified patterns across platforms requires a general framework within which virtually any kind of data can be processed in a cross platform environment: that of a database. Data (including patterns) from one platform are then imported into this database, where these patterns are further processed to uncover further properties, then the patterns can be exported to another platform, including the one the data originated from. This contribution aims at introducing a new methodology and a tool implemented as a web-based service for these purposes.

## 1. Introduction

Mixed methods research represents “a new movement, or discourse, or research paradigm (with a growing number of members) that has arisen in response to the currents of qualitative research and quantitative research” (Johnson et al., 2007, p. 113). Many researchers do not mix qualitative and quantitative approaches in optimal ways, according to Powell et al. (2008), but qualitative techniques can be used to enhance the development of quantitative instruments, as scales, and vice versa (Collins et al., 2007). Their potential is very broad, and includes instrument fidelity, “maximizing the appropriateness and/or utility of the instruments used, whether quantitative or qualitative” (Onwuegbuzie et al., 2010, p. 57).

The aim of our work goes in this direction and focuses on some concrete methodological issues which are related to post-processing techniques of hidden patterns detected in time-aligned data. The development is motivated by the research questions of the HuComTech project (Hunyadi et al., 2016a,b) to explore the temporal structure of multimodal communication based on an annotated spontaneous speech corpus. For *T-pattern* detection (Magnusson, 2000), we used the *Theme 6 Full* (Pattern Vision, 2018) program. Although it has several excellent functions for post-analyzing the resulting hidden patterns of behavior (e.g., visualizing pattern structures, filtering results by event types and quantitative attributes, separating markers, predictors etc.), it cannot be really required to satisfy all the needs of every custom research. If any special demands arise which cannot be handle within the tool, one can achieve other ways of processing by using the built-in option of data extraction. After this first step, we have to face with another problem. The conversion of behavioral data (as the input of detection) is supported by ELAN (Wittenburg et al., 2006), but there is no available tool for importing the resulting T-patterns back into ELAN or other annotation tools in which the data was originally created, or into any other framework supporting further analysis. However, it would be beneficial to see the patterns as “part of the data” aligned with the source media (audio and video files) and explore their uncovered properties and interrelations. Another issue arises if we need to apply T-pattern analysis separately on different sources of behavioral data (without creating “multisample files” in *Theme*) but we are also interested in the common patterns that originated from different sources. These common patterns cannot be easily identified, because in *Theme*, the *uniqueness* of T-patterns is determined within one single source of data (where they were detected), and it can result in multiple instances of the very same pattern type.

The current methodological contribution aims to demonstrate a practical solution for the above mentioned issues, using PHP, Javascript and MySQL (Widenius and Axmark, 2002) as development tools for implementation. The outcome is a web-based interface which we developed for re-organizing the outputs of T-pattern detection in a relational database where their management is much more effective and transparent, and they can also be exported into ELAN or other tools for further analysis. Similarly to the *Subjectbook* project (Taamneh et al., 2016), it is a crossplatform interface for exploring and analyzing behavioral data, and in the narrower context of our particular field of interest, it can be considered as a supplementary component of the *Data Exchange Platfrom* (Pattern Vision, 2019). *Theme* users already have a software and an exchange annotation format (Schmidt et al., 2009) to import behavioral data for T-pattern detection from various data collection tools, and now, we would like to facilitate access to data in the opposite way: import back the resulting T-patterns for post-analysis.

In the next section 2, we introduce some sample data (exported from *Theme* projects) which we used for development purposes. Then in section 3, the main issues of data analysis are discussed. It is followed by sections 4, 5, where we introduce the details of our methodology and the results of development using explanatory examples.

## 2. Research Material

Two sample resources were used for development purposes. Both of them were collection of T-patterns exported from *Theme* projects based on the data of the HuComTech corpus^1^. All annotations were available in EAF and Praat TextGrid (Boersma and Weenink, 2019) format and they were converted to *Theme* using a custom Praat script. Since our contribution aims at demonstrating a special methodology of data processing without going into details about the output itself, we only mention the relevant characteristics of the development data.

The HuComTech multimodal corpus contains ~50 h of behavioral data based on annotated audio and video recordings of spontaneous dialogues. The interactions were performed by Hungarian native speakers (between the age of 18 and 30) using two types of interview scenarios for every participant (111 in total): a simulated job interview and a subsequent informal conversation. Beyond the transcribed speech, the annotation of the corpus follows various perspectives and modalities including time-aligned labels for nonverbal gestures, expressed emotions, speech acts, prosody and syntax. However, in the actual experiments, we limited the set of labels (*event-types* in *Theme*'s terminology) to some specific ones (see details in the subsections below). The imported samples are also reduced to one formal and one informal interview from 2 different speakers (4 datafiles in total). For making the detection even faster and the amount of resulting patterns more manageable, we also exlude *univariate patterns* and set the *maximum search level* to 3 with an extremely rigorous significance level (*p* < 0.000005) to estimate the probability that the patterns discovered were not the result of chance.

### 2.1. Experiment 1: Structure of Turn-Taking

In the first experiment, the input of T-pattern detection was limited to the labels of *turn-taking* in the formal and informal conversation. Following a basic principle of segmentation, we only have four types of events: (1) speech of the interviewer, (2) speech of the interviewee, (3) silence, (4) overlapping speech. They were represented as four *actors* (*agent*, *speaker*, *overlap*, *silences*) with one possible corresponding event (*speech* for the first three and *silence* for *silences*) in the VVT file (the special template format of *Theme* used for declaring the type of data to be imported). Since the events of input sequences were extracted from time intervals of the original annotations, they were also extended with (*b*)*egin* and (*e*)*nd* specifications (for instance, “b, agent, speech” means that the interviewer starts speaking and “e, agent, speech” means that the interviewer ends speaking). Despite the applied constraints (maximum search and significance level), the T-pattern detection resulted in 6,516 patterns (including redundant ones) with 115,683 occurrences in the 4 datafiles.

### 2.2. Experiment 2: Topic Shift and Prosody

In the second experiment, prosodic labels (namely, silences and intonation shapes of speech) and topic shifts (the beginning of those speech acts where the interviewer asks a new pre-planned question) were imported into *Theme*. Topic shifts are represented as topic boundaries under the category name of *topics* (e.g.,: “b, topics, topic” means that a new topic has started). For silences, the same representation was used as in the previous experiment. Segments of *intonational events* (the term was adapted from Taylor, 2000) were generated by a rule-based algorithm (Szekrényes, 2015) which was developed for automatic annotation of stylized shapes of intonation. This labeling method was applied to the speech of every related actor (*agentf*0*mov*, *speakerf*0*mov*, *overlapf*0*mov*- overlapping speech was considered as a “third speaker” here) using five possible categories: *rise*, *fall*, *level*, *ascending* and *descending*. The T-pattern detection resulted in 22,092 patterns with 229,019 occurrences in the 4 datafiles.

## 3. Actual Issues of Data Analysis

In this section, two issues of data analysis are discussed as the motivation of the current methodological contribution:

1. In a Theme project, we cannot easily find out which patterns are detected in more than one datafile, because their uniqueness is interpreted locally (within one datafile) and not globally (within the whole project). However, it is very likely that some of the patterns are shared among individual files as different instances of the same pattern type.
2. Beyond spreadsheet and simple text processing programs, there is no available tool for post-processing the exported T-patterns in an effective way. However, it would be beneficial to have a cross-platform framework to manage the output data supporting conversion to other tools including those ones where the input data was originally created.

### 3.1. T-patterns From Various Sources of Data

The first issue we mention here is particularly important in case of *Theme* projects with a number of various and categorizable sources of data. For instance, in the HuComTech corpus, the recorded conversations can be separated by both speakers and the type of the interview scenario (formal and informal). Technically, they are *independent variables* and the related samples may contain various temporal patterns. The question is how to find the “common” T-patterns that are detected in more than one source of data given that they are interesting from any perspective.

**Figure 1:**
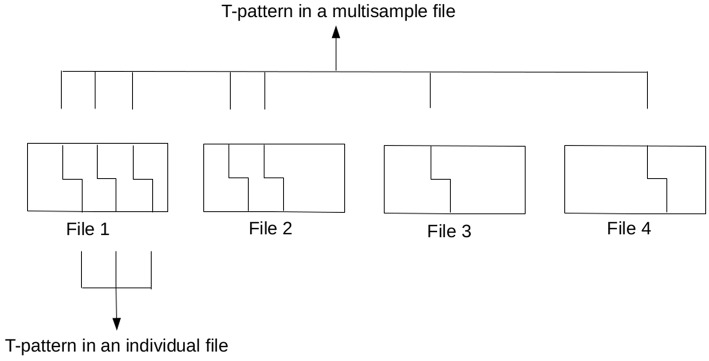
Difference between detecting a T-pattern in an individual and a multisample file (the *minimum occurrence* parameter was set to 3 in this example).

If one uses a so-called *multisample file* option available in *Theme* by merging data from various sources based on one of the independent variables (e.g., every informal conversation of the HuComTech corpus), the resulting patterns will show us what types of patterns are repeated if we considered individual sources of data as subsequent parts of the global timeline of observation. Although it is a very interesting and adequate research question, it must be taken into account that T-pattern detection can collect such patterns in a multisample file, which were not originally repeated (and would not have been detected) in some of the individual datafiles (as can be seen in Figure 1) or it is also possible that some of the resulting T-patterns would not have been detected in any file. Of course, it is not necessary an issue, moreover, this is the main benefit of using the method. However, in certain cases, we would like to reword the question exactly interested in those types of *common* patterns which are similarly repeated in multiple sequences and/or different types of data. For instance, we would like to know about those patterns which can be found in both the formal and the informal conversations of HuComTech corpus. Actually, the same type of information is required if we are interested in the individual characteristics of a certain dataset (based on a given independent variable) focusing on those patterns which *cannot be found* in other sources of data.

If one exports T-patterns from a *Theme* project consisting several datafiles (they can also be multisample files), the *uniqueness* of patterns must be interpreted *locally*, i.e., they are unique within the file where they were found. If the same pattern (having the same structure of event types described by the corresponding *patstring* attribute) occurs in more than one datafile, the different instances (cases of detection) are represented as different patterns. As can be seen in the exported *patstring.txt* file (see Table 1), every pattern is identified by two kinds of attributes: the source of data (*dataname*) and an identifier (*id*). The last one is not unique for the whole project, but it distinguishes patterns only within the particular source of data. Therefore a specific pattern structure is functionally dependent on both the *dataname* and the *id* attributes in the logical model of this data collection. Our goal is to replace this logic with a *global scope* of T-pattern identification where “uniqueness” is interpreted *globally* (for the whole project) ignoring multiple cases of detection by referring to a list of abstract event-structures wherever any of them was detected.

**Table 1:** Identification of patterns using *dataname* and *id* attributes in *patstring.txt*.

**Dataname**	**Id**	**N**	**Length**	**Level**	**N_actors**	**N_switches**	**Patstring**
f006mc22f	58	21	2	1	1	0	(b,agentf0mov,level e,agentf0mov,rise)
f006mc22f	59	9	2	1	1	0	(b,agentf0mov,rise b,agentf0mov,descending)
f006mc22f	60	46	3	2	1	0	(b,agentf0mov,rise (e,agentf0mov,rise b,agentf0mov,fall))
f006mc22f	61	13	3	2	1	0	(b,agentf0mov,rise (b,agentf0mov,level e,agentf0mov,rise))
f006mc22f	62	8	2	1	1	0	(b,agentf0mov,rise e,agentf0mov,descending)
f015mv26f	58	26	3	2	1	0	(b,agentf0mov,rise (e,agentf0mov,rise b,agentf0mov,fall))
f015mv26f	59	4	2	1	1	0	(b,agentf0mov,rise e,agentf0mov,ascending)
f015mv26f	60	4	2	1	1	0	(b,agentf0mov,rise e,agentf0mov,descending)
f015mv26f	61	29	2	1	1	0	(b,agentf0mov,rise e,agentf0mov,fall)
f015mv26f	62	7	2	1	1	0	(b,agentf0mov,rise e,agentf0mov,level)

*The samples are from Experiment 2*.

### 3.2. Post-processing of T-patterns

To implement the above mentioned way of data representation, we need a post-processing method and an external database where the exported data can be imported and re-organized using a suitable logical model, then custom database queries can be constructed to uncover further properties (e.g., the number of datafiles where the pattern was detected) and interrelations of the resulting T-patterns and their physical locations. The framework also has to support further exportation of data into existing annotation tools where the input of T-pattern detection was originally created. We used ELAN and Praat for video and audio annotation. These tools are very popular, widespread, free and open source software, moreover, we can export data into several other tools from ELAN, therefore it seemed to be the most ideal destination for data export. If one uses time-aligned labels associated with media files as an input to T-pattern detection (and this is a very common case in behavioral science), there are reasonable advantages to associate the resulting patterns as well. Having T-patterns aligned with annotated media significantly expands the possibilities of post-analysis by exploring pattern locations using speech or image processing algorithms for media, structured queries in annotations and even qualitative methods to interpret and gain a deeper understanding of the specific context of repeated event-structures. In these types of post-analysis, we can also include such kinds of data which were previously ignored from T-pattern detection (e.g., the textual transcriptions in case of the HuComTech corpus). After the exportation of a *Theme* project, all the necessary information is available (the structure of resulting T-patterns with their locations), we only have to process and represent the exported data in an appropriate way.

## 4. Methodology

**Figure 2:**
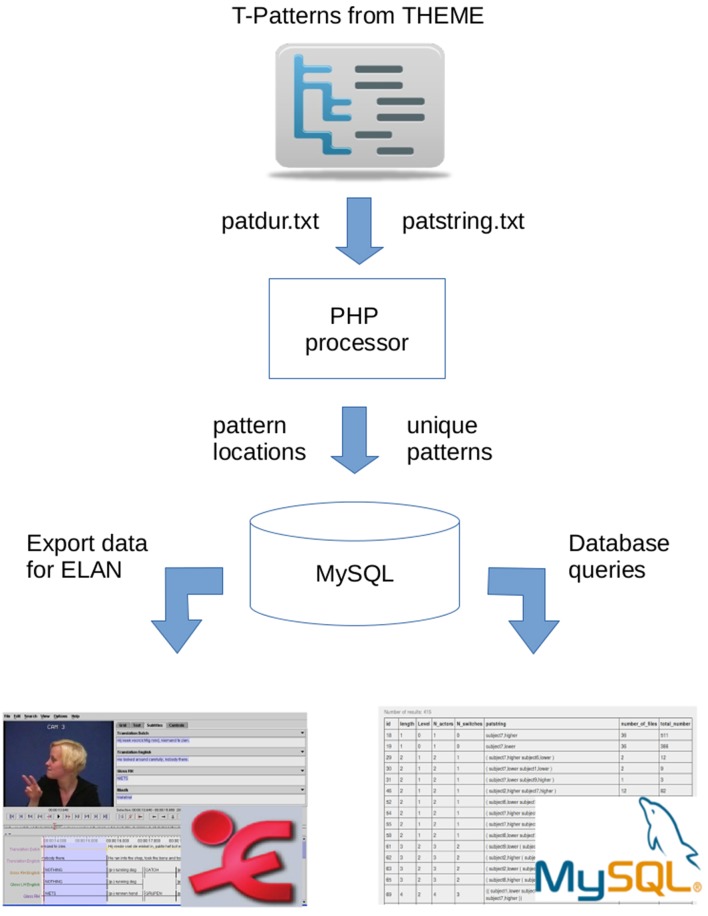
The overview of data flow and the main components of the interface.

Since we wanted to implement a platform independent solution for handling the above mentioned issues, a web-based interface was developed where the exported *Theme* projects can be uploaded and managed using a simple web browser. For the development, standard and open source W3C tools and technologies were used such as CSS bootstrap, PHP, MySQL (community version), Javascript, Ajax (Zakas et al., 2006; Zhang et al., 2008; Dong et al., 2011). Figure 2 shows the main components of the processing chain starting with a *PHP processor* which is to convert and import data into a *MySQL database*. In the subsections below, we describe the complete flow of data processing in details.

### 4.1. Converting, Re-indexing and Filtering Unique T-patterns

The exportation of a *Theme* project results in two plain text files using tab-separated table format. The first table (*patstring.txt*) contains special attributes of T-patterns: the source of detection (*dataname*), a “unique” index of pattern (*id*), the number of occurrences in the source of detection (*N*), the number of events the pattern contains (*Length*), the search level where the pattern is detected (*Level*), the number of event categories associated with the pattern (*N*_*actors*_), the frequency this category subsequently changes in the pattern (*N*_*switches*_), and finally, the complete string of events the pattern consists of (*patstring*). The other exported table (*patdur.txt*) lists the exact locations (source of data and temporal properties: *starttime*, *endtime* and *duration*) of every T-pattern referring to both the *dataname* and the *id* attributes (used as *complex keys* in the first table) to link a specific location to a particular pattern.

**Figure 3:**
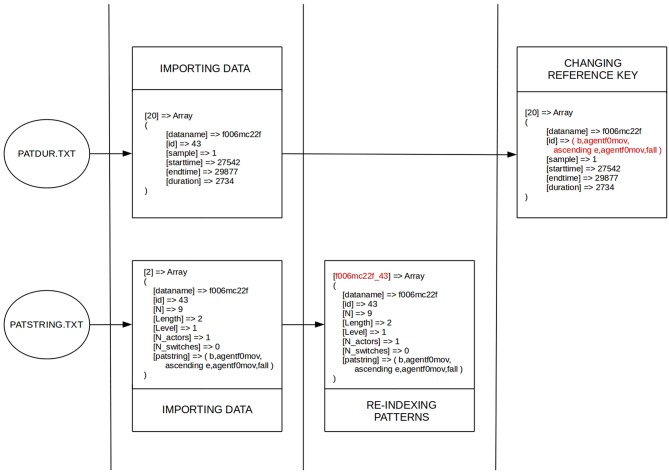
First steps of data processing: working with multidimensional arrays.

**Figure 4:**
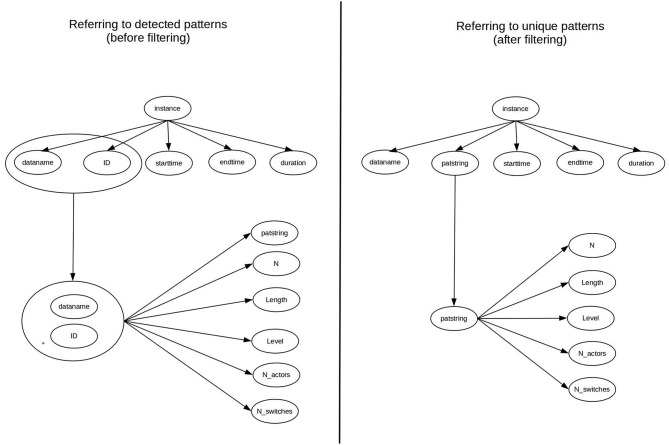
Data dependency graph of the exported data before and after filtering T-patterns.

After uploading the input data to our interface, a PHP script processes these tables and converts the data into two multidimensional arrays (one for T-patterns from *patstr.stx* and another one for their locations from *patdur.txt*) using column names for indexing the attributes of array elements. As can be see in Figure 3, each element of array of T-patterns are re-indexed in the next step using the combination of its key attributes (*dataname* and *id*) instead of the original numeric indexes. It makes the connection between T-patterns and their locations more transparent for processing algorithms. Based on the new indexes, we can easily associate pattern locations with the corresponding T-pattern, therefore the value of the *id* attribute in the array of pattern locations can be replaced with the value of *patstring* attribute of the referenced pattern. It is needed before we eliminate redundancy in the array of T-patters (in the next step) by filtering elements based on the uniqueness of the *patsrting* attribute. After the filtering, *id* and *dataname* attributes are not further needed for the T-patterns and they cannot be used as reference key in pattern locations either, since there is a chance that the corresponding array element does not exists any more. As can be seen in Figure 4, every pattern instance (with the data of an exact location) refers to a unique pattern using the *patstring* attribute at this stage of processing.

**Figure 5:**
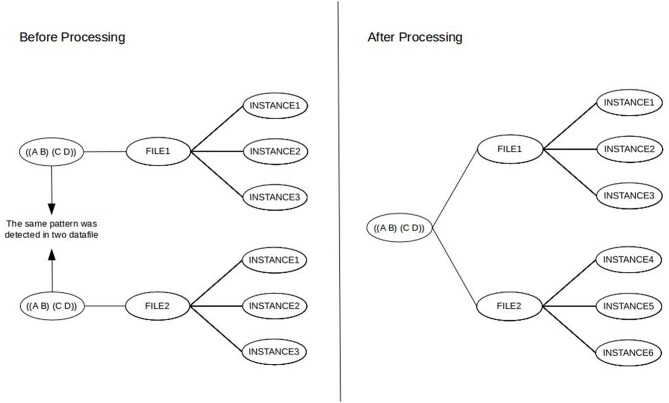
The original **(Left)** and the resulting global scope **(Right)** of T-pattern identification.

As can be seen in Figure 5, the issue of multiple pattern instances (the first issue in section 3) is already solved by the above data normalization as a result of the global scope of T-pattern identification. However, we also need to implement this logical model as a real database.

### 4.2. Creating the Relational Database

**Figure 6:**
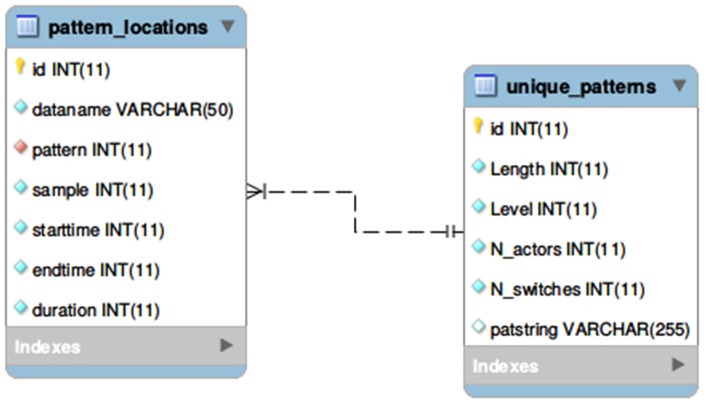
Representing the result of T-pattern detection in a relational database.

For creating the database, we applied a standard relational data model (Codd, 1990) supported by the SQL Data Definition Language and the MySQL program. Even though, there are other open source platforms also available to implement a similar database, we choose our logical model as a typical example for 1:*n* type relation of entities (between patterns and their locations) to be best represented in a relational schema. Following other, NoSQL paradigms (Pokorny, 2011), such as the document-oriented concept of *MongoDb* would not have been really well-founded four our purposes. Therefore after having the final state of the two multidimensional arrays, all data are inserted into a MySQL database using two relational tables for *Theme* projects preserving every attributes of the entities. As can be seen in Figure 6, the *unique-patterns* table contains the result of filtering (the unique T-patterns detected in the project), while the *pattern-locations* table provides information about their physical instances occurring in one or more datafiles.

The *id* attribute has an auto increment *integer* value in both of the tables. They are generated during insertion and used as *primary key* for database records. The *pattern* attribute of the *pattern-locations* table is a *foreign key* which refers to the primary key of unique patterns. During inserting data into *pattern-locations*, we used an embedded query to find the corresponding index that can be used as *foreign key* instead of the value of *patstring* attribute which would cause redundancy. In case of huge amounts of T-patterns, importing a new project is a quite long procedure, but it is managed by a background PHP process on the server (logging progression into a JSON file), therefore the user can perform other operations on the site in parallel (e.g., run queries in other projects) and they can also check the actual state of importation in their browser. In the resulting database, one can store and query the data using various ways and communication protocols (PHP, node.js, Python etc.). In our framework, we use the database to explore *Theme* projects via SQL queries and as an intermediate storage for exporting the data to other platforms as a solution for the second issue we mentioned in section 3.

### 4.3. Exploring Imported Projects

**Figure 7:**
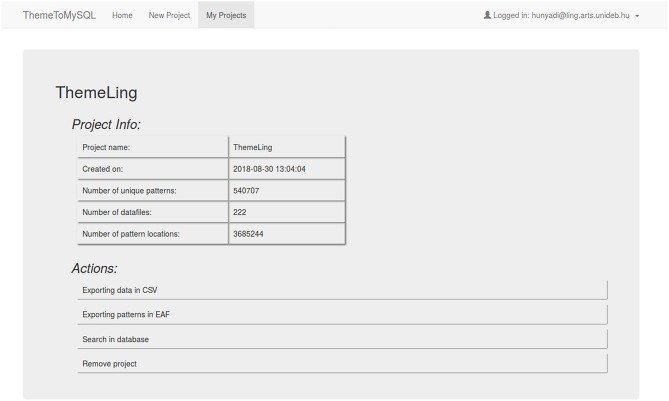
Overview of an imported *Theme* project: general statistics and management possibilities.

Once the project is imported to a MySQL database, it can be easily managed with a simple menu-system (see Figure 7). In addition to dump export of processed data (EAF and CSV format are supported currently), one can also explore the imported project within the interface by composing SQL queries on an HTML form. Users can add various conditions to the query and filter the resulting table by any attributes of the given T-patterns.

**Figure 8:**
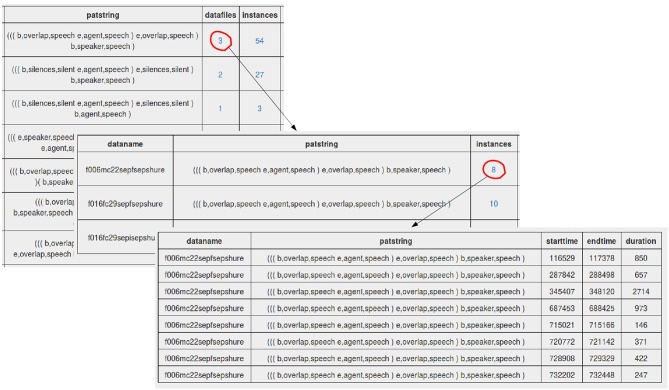
Sequence of database queries: based on the resulting table of T-patterns, one can check the datafiles for a particular pattern, then the exact locations (timing properties) inside that file.

Unique T-patterns can be queried using various filters. In the resulting table, besides the usual attributes of patterns (*N*_*actors*_, *N*_*switches*_ etc.) one can check how many physical instances a specific T-pattern has and in how many datafiles. As can be seen in Figure 8, these datafiles and the exact locations can also be queried by clicking on the respective number in the selected record.

### 4.4. Exporting T-patterns Into EAF

Since EAF (the acronym means *ELAN Annotation File*) is an XML-based annotation format, the exportation method itself is quite simple: the required data (containing the structure of the selected T-patterns and the exact locations of their instances) is queried from MySQL, then a PHP script generates the XML file using the DOM Document parser. In the resulting EAF files every T-pattern is represented as a unique *annotation tier* where the annotation labels contains the pattern structure in textual format using brackets to express the hierarchy of events.

**Figure 9:**
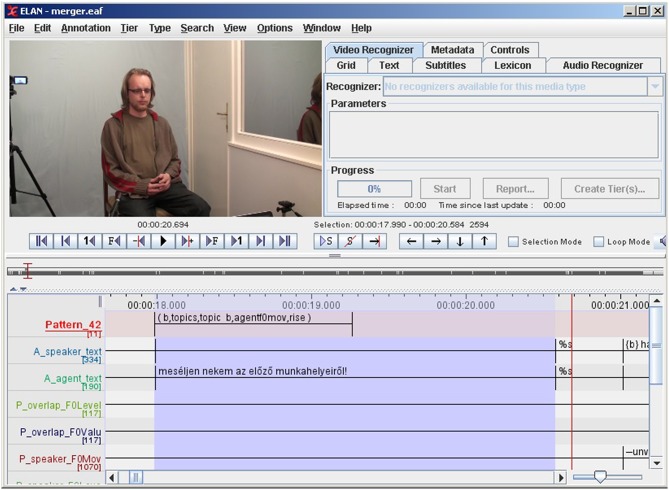
Representing T-patterns in ELAN. In this example, the exported EAF was previously merged with the original annotation file. The first annotation tier displays the instances of *Pattern*_42_.

As can be seen in Figure 9, ELAN displays these annotation tiers as parallel timelines aligned with the media file (the original source of observation) where we can locate the occurrences of T-patterns as intervals of time. We can also merge the output with the original annotation files (ELAN has a built-in function for that) that makes the post-analysis more efficient, since we have the whole context including those annotated events (or textual transcriptions of speech) which are not part of the pattern but co-occur with it. Unfortunately, a dump export of the internal temporal structure of T-patterns (the times of events that the pattern contains) is not supported by the current version of *Theme* and neither ELAN supports labels without any duration. However, Praat could display them in a special annotation tier (it is called “PointTier” in Praat), and one of our future plans is to create an alternate XML structure, as soon as the exportation is solved from *Theme*.

## 5. Results and Availability

The results of this development were already presented during the 10th meeting of MASI (Research Network on Methodology for the Analysis of Social Interaction, University of La Laguna, Tenerife, Spain, September 13-15, 2018.), but in the meantime, we made a demo interface available^2^ for the community of *Theme* users. The project is published under the name *ThemeToMySQL* referring to that component of the interface where projects are imported. Alternatively, it could be called “ThemeToELAN” as well, since we made the conversion bidirectional between ELAN and *Theme*. In any case, the central part of what we are offering now is the database which can serve as the basis for any further kind of exportation.

Because of the limited computer capacity (we currently have 2 CPU cores and 16 Gbyte RAM available on the server), the demo interface also has some limitation on the allowed number of importable projects and the size of data files. But if the user would like to install a custom version using their own resources, the source code can be freely downloaded from a GitHub repository^3^. The interface was tested with several *Theme* projects including a very complex one in which the T-patter detection resulted in 967,447 patterns with 3,685,245 occurrences based on 20 datafiles from the HuComTech corpus, but here, we only report some results from the two experiments described in section 2. It has to be emphasized that these results are based on a very small amount of data. As explanatory examples, they only serve demonstration purposes.

### 5.1. Experiment 1: Structure of Turn-Taking

In a former research (Szekrényes and Kovács, 2017), we trained Deep Neural Networks to distinguish the type of interview (formal or informal) based on similar sequences of turn-taking resulting in surprisingly high accuracy (81.5%) of automatic classification. Therefore it seemed very interesting to test whether we can evince some differences between the two interview types based on the results of T-pattern detection.

After processing the project on the interface, the total number of unique T-patterns was reduced to 4,952. Since overlapping speech can be a possible distinctive feature between formal and informal interviews, let us query the most frequent T-patterns which contain at least 3 actors including *overlap*. The results can be seen in Table 2.

**Table 2:** Resulting table of the first query containing T-patterns (only the relevant attributes are preserved here).

**Patstring**	**Datafiles**	**Instances**
((e,agent,speech (b,speaker,speech e,speaker,speech)) b,overlap,speech)	4	102
(e,overlap,speech ((b,speaker,speech e,speaker,speech) b,agent,speech))	4	98
((e,speaker,speech (b,agent,speech e,agent,speech)) b,overlap,speech)	3	98
(e,agent,speech ((b,speaker,speech e,speaker,speech) b,overlap,speech))	4	93
((e,overlap,speech (b,speaker,speech e,speaker,speech)) b,agent,speech)	4	90
((e,overlap,speech (b,agent,speech e,agent,speech)) b,speaker,speech)	4	89
((b,agent,speech e,agent,speech) (b,overlap,speech (b,speaker,speech e,overlap,speech)))	4	87

The last record appears to be interesting, since this pattern structure expresses a kind of impolite way of turn taking (the speaker takes turn by using overlapping speech). However, we have to be careful with this interpretation, because we cannot surely know if these events (agent, overlap, speaker) are subsequent ones (unless we check the patterns in ELAN). Finally, we can also query the distribution of this pattern in formal and informal interviews. As can be seen in Table 3, this particular pattern is much more frequent in the informal ones.

**Table 3:** Resulting table of the second query showing the distribution of the pattern's locations.

**Dataname**	**Type**	**Instances**
006mc22sepf	Formal	8
f006mc22sepi	Informal	38
f016fc29sepf	Formal	6
f016fc29sepi	Informal	35

### 5.2. Experiment 2: Topic Shift and Prosody

In case of this experiment (containing 20,741 unique patterns), those T-patterns could be interesting where both topic shifts and prosodic events are presented in the pattern's structure expressing possible prosodic markers of topic change. Therefore we should concentrate on T-patterns which contain *topic shift* event with another actor occurring across all the datafiles. Unfortunatelly, there is no such a pattern in our dataset. However, if we set the “minimum number of datafiles” threshold lower, the resulting table contains a pattern “(*b, topics, topicb, agentf*0*mov, rise*)” which occurs in all the formal interviews but none of the informal ones.

## 6. Summary

Here, we introduced a new methodology for post-processing T-patterns exported from the *Theme* program. The project has two important outcomes highlighted here:

1. Using a relational database model, the interrelations of T-patterns and the input source of data became more transparent and manageable,
2. In support to mixed method research, our interface makes T-pattern analysis more flexible and externalized by offering tools where the patterns can be associated with the source media and the original context of behavioral data.

The method was implemented as a web-based interface which is freely available for research purposes. Any kinds of *Theme* projects can be imported, explored or converted into XML-based annotation files. For further development, we have several future plans including: (1) optimizing database-import of T-patterns, (2) making database queries more customizable, (3) supporting more data formats for exportation.

## Author Contributions

The author confirms being the sole contributor of this work and has approved it for publication.

## Conflict of Interest Statement

The author declares that the research was conducted in the absence of any commercial or financial relationships that could be construed as a potential conflict of interest.

## Footnotes

^1^“HuComTech Corpus” from The Language Archive: https://tla.nytud.hu/MTARIL/01-0003-0000-0000-0000-1@view

^2^https://altnyelv.unideb.hu/ThemeToMySQL/login.php

^3^https://github.com/szekrenyesi/ThemeToMySQL
